# Using mHealth to Improve Timeliness and Quality of Maternal and Newborn Health in the Primary Health Care System in Ethiopia

**DOI:** 10.9745/GHSP-D-20-00685

**Published:** 2021-09-30

**Authors:** Zeleke Yimechew Nigussie, Nebreed Fesseha Zemicheal, Gizachew Tadele Tiruneh, Yibeltal Tebekaw Bayou, Getnet Alem Teklu, Esubalew Sebsibe Kibret, Kristin Eifler, Sarah E. Hodsdon, Dessalew Emaway Altaye, Leona Rosenblum, Yeshiwork Aklilu Getu, Zinar Nebi, Ephrem Tekle Lemango, Eyob Kebede, Wuleta Aklilu Betemariam

**Affiliations:** a JSI Research and Training Institute, Inc., Addis Ababa, Ethiopia.; b JSI Research and Training Institute, Inc., Boston, MA, USA.; c Ministry of Health, Addis Ababa, Ethiopia.

## Abstract

The Last Ten Kilometers 2020 Project (L10K 2020) designed a strategy for piloting, implementing, and scaling a mobile health (mHealth) digital solution to improve the quality of community-level maternal and child health service delivery in Ethiopia. L10K 2020 first conducted a landscape assessment to design a context-appropriate smartphone-based mHealth solution for the Health Extension Workers and tablets for their supervisors and the midwives managing the same clients at the health center level. These applications included multiple modules and packages including client registration and appointment management; follow-up and notifications; digital job aids for each of the maternal and child health program packages (for Health Extension Workers only); and referral and client tracking systems.

Findings from the process evaluation of the mHealth app usage and user experience indicated that the application was user-friendly and facilitated real-time information exchange, defaulter tracing, referral, and feedback systems. It improved the timely identification and registration of pregnant mothers. Adherence to treatment protocols also increased in all domains across the pregnancy continuum of care.

L10K 2020 has developed a user-friendly model for implementing mHealth solutions at the community level through stakeholder engagement across levels when developing, testing, and deploying the applications, which was critical to effectively cultivating ownership as well as skills and knowledge transfer at all levels. To replicate and scale this model, context-based scoping, resource analysis, and mapping are essential to determine the infrastructure, cost, time, risks, and key stakeholders involved throughout the implementation of the intervention. During implementation, vigilance in consistently mitigating the challenges related to mHealth infrastructure, such as mobile data capacity, electricity, smartphones and tablets, solar chargers, and internet connectivity, is critical for continued success.

## Abstract (precis)

The use of mobile health (mHealth) in Ethiopia’s primary health care system offers a potential solution to improve timeliness and quality for maternal and newborn health care services. It is user-friendly and fosters communication between health care workers and health extension workers to provide quality services across the pregnancy continuum of care.

## Teaser

**Key Findings:**

- The Last Ten Kilometers 2020 Project (L10K 2020) designed a user-friendly mobile health (mHealth) solution that bridged communication gaps between health care workers and health extension workers and improved quality services for women across the pregnancy continuum of care delivery in Ethiopia.
- Findings from routine monitoring and usability assessment showed that the mHealth application facilitated real-time information exchange with supervising health facilities in the primary health care system, as well as timely identification and registration of pregnant women, thereby increasing uptake of maternal health services across the continuum of pregnancy, childbirth, and postnatal stages.

**Key Implications:**

- Engaging with health sector stakeholders at all levels when developing, testing, and deploying the mHealth solution can effectively cultivate ownership and ensure skills and knowledge transfer at all levels.
- Using a cocreation strategy to develop an mHealth solution will help ensure the smooth handover and scalability of the model and build local capacity.
- Vigilance in consistently mitigating the challenges related to mHealth infrastructure, such as mobile data capacity, electricity, and internet connectivity, will reduce usage disruption, and bolster users’ confidence in the app’s capabilities.

## BACKGROUND

Mobile health (mHealth) refers to the use of wireless technology and devices (smartphones and tablets) to enhance access to information and improve the delivery of basic health care services.^1^^,^^2^ Over the past 3 decades, a range of digital technologies have emerged for sharing and generating health and medical information, and the fields of mHealth and digital health have expanded globally.

Real-time access to reliable and accurate information to deliver consistent and high-quality health care is in high demand.^3^ Globally, the application of mHealth solutions in the public health sector has contributed to improvements in the delivery of quality health care.^4^^,^^5^ mHealth applications are widely acknowledged as a way to transform how clients and health providers exchange health information,^4^ and they present the opportunity to improve the quality and timeliness of maternal and child health services and strengthen referral linkages, particularly in under-resourced health systems.^4^^,^^6^^–^^11^

Ethiopia achieved most of the Millennium Development Goals for health^12^ through well-coordinated and extensive efforts made by the government, community, and implementing partners through the health extension program (HEP) and the expansion of primary health care services. The government and its partners have prioritized mobile technology as a potential solution to revitalize Ethiopia’s HEP and the country’s overall primary health care system.^6^^,^^13^ In collaboration with the Ministry of Health (MOH), the Last Ten Kilometers 2020 Project (L10K 2020), implemented by JSI Research and Training Institute, Inc., designed an mHealth strategy to complement the existing MOH interactive voice response (IVR) system. This system allows Health Extension Workers (HEWs) to call and record information and send/review their activity reports to a centralized database system.

The primary aim of the L10K 2020 initiative was to improve delivery, timeliness, and quality of maternal and child health services by leveraging existing mHealth technology to support service provision and strengthen linkages within the primary health care units (PHCUs) (i.e., health centers and their catchment health posts). The specific objectives included the following:

1. Improve timeliness and coverage of reproductive, maternal, newborn, and child health (RMNCH) services
2. Improve the quality of RMNCH services
3. Improve referral care (number/proportion of cases referred from health post level to health centers) for RMNCH services by linking the health extension application with health center focal person application and by leveraging information communication technology (ICT)

The initiative aimed to improve delivery, timeliness, and quality of maternal and child health services and to strengthen linkages within PHCUs.

Once the mHealth initiative was designed in line with MOH’s vision, policies, and strategies, L10K 2020 designed incubation sites for learning to field test the initiative in Mirab Azernet woreda of Southern Nations, Nationalities, and People’s Region (SNNPR) followed by expansion to other 3 rural districts: Dembecha (Amhara), Shebe Sombo (Oromia), and Werai Leke (Tigray).

This article outlines L10K 2020’s experience introducing the mHealth strategy for improving RMNCH care. Specifically, it:

1. Describes the mHealth solution design and implementation process
2. Documents the mHealth solution usability and usage across the continuum of care
3. Examines the role of the mHealth solution in enhanced RMNCH service delivery
4. Describes the contribution of the mHealth solution to the electronic community health information system (eCHIS)
5. Documents lessons learned and challenges

To organize this article, we reviewed all project documents including the mHealth program plan, reports, findings from routine monitoring, and the implementation process evaluation. Process evaluation included a review of design, scoping, development, deployment, troubleshooting, application usage monitoring, and feedback systems used in the implementation of mHealth programs. We also reviewed the support and engagement of the health sector.

## MHEALTH SOLUTION DESIGNING PROCESS

### Landscape Assessment

**FIGURE 1:**
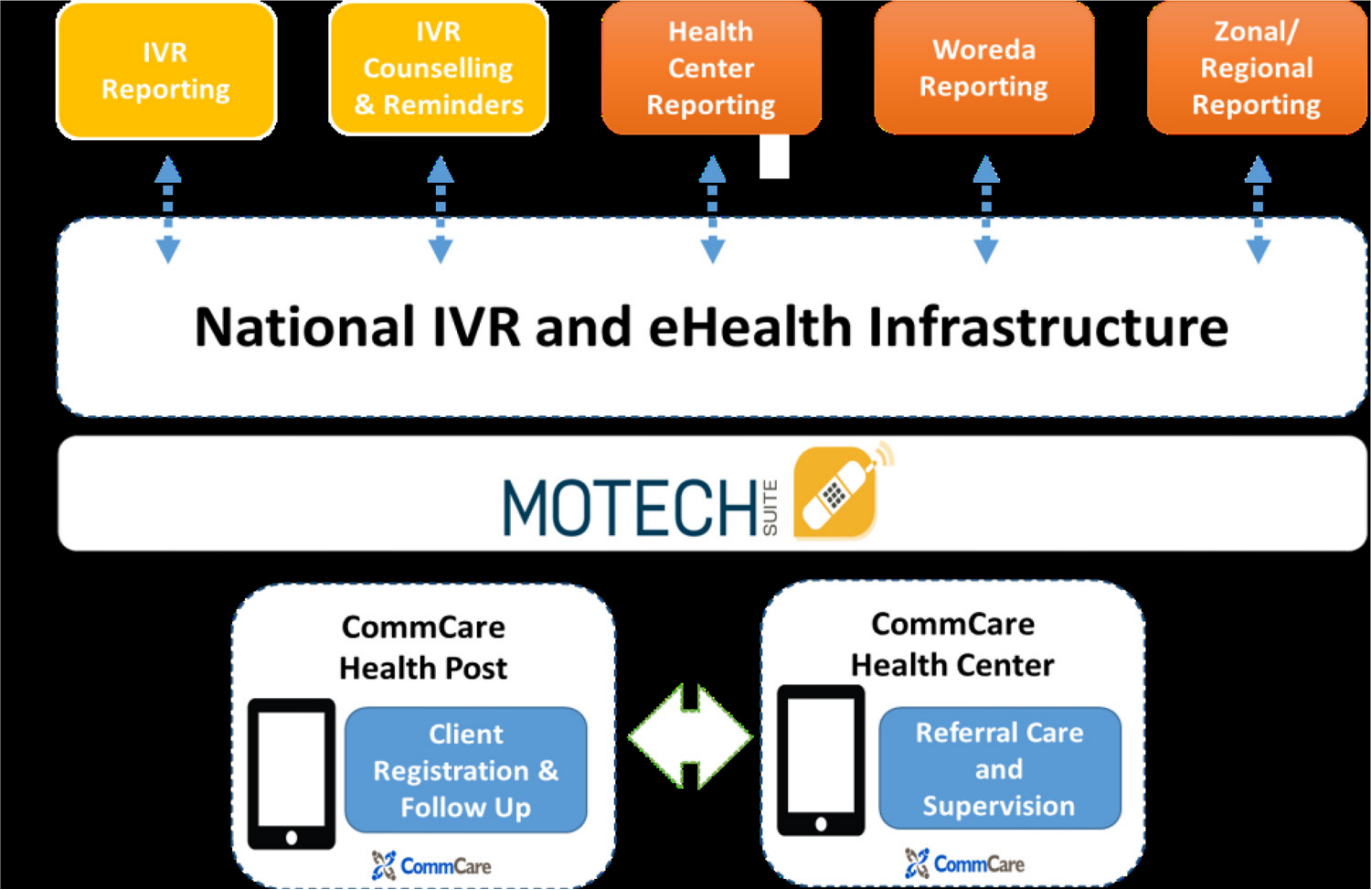
Proposed Technology Architecture for mHealth Initiative in Ethiopia Abbreviations: IVR, interactive voice response; mHealth, mobile health.

In 2015, L10K 2020 conducted a landscape assessment to investigate the local context and to propose a feasible technological architecture (Figure 1) aligned with L10K 2020 program objectives and opportunities as related to MOH objectives. The assessment covered the ICT infrastructure of the pilot sites, including mobile penetration for HEWs; mobile and data network coverage of health centers, health posts, and villages; availability of electricity or solar power for health centers and health posts for charging of devices; review of the national strategies related to eHealth and the key components of the health information system, including the community health information system, electronic health management system, the District Health Information System (DHIS2), and the MOH IVR system. Core strengths and challenges in the existing system were identified through interviews, workflow observation, and document review. Midwives, HEWs and their supervisors, health information technicians, and program managers in the primary health care system were interviewed in the pilot woredas. The assessment findings showed that all the health centers in the proposed intervention areas of mHealth had mobile networks with mobile data networks and access to electric power. While 91% of the health posts had both mobile network and mobile data (internet) network coverage, only 25% and 12% of them had electric and solar power coverage, respectively. The findings of the landscape assessment were used as inputs to set the requirements and recommendations for the project implementation.

### mHealth Solutions Designing

Based on the findings of the landscape assessment and prior in-country use of the technology, CommCare* was identified as an appropriate platform for leveraging existing national efforts such as IVR and the electronic health information system infrastructure (Table 1). This platform could also support future MOH goals. Through a series of discussions, MOH agreed on the use of CommCare as a platform for the initiative with the expectation that implementation would complement the existing IVR system, support the national eHealth strategy, and be interoperable with the national health management information system (HMIS).

**TABLE 1.** Summary of Objectives of the mHealth Initiative in Ethiopia With Corresponding Technology Components

	**Health Objectives**	**Technology Components**
1.	Improve timeliness and coverage of RMNCH services provided by health posts	Leverage electronic platform for automated reminders and follow-up notifications and alerts
2.	Improve the quality of RMNCH services provided by health posts	Leverage electronic platform for mobile electronic job aids (e.g., checklists) for Health Extension Workers
3.	Improve referral care for RMNCH clients to health centers	Leverage electronic platform for improved data and referral workflow

Abbreviation: RMNCH, reproductive, maternal, newborn, and child health.

The CommCare platform leveraged existing national efforts and could also support future MOH goals.

The electronic platform we chose is interoperable with the district health information software (DHIS2) platform on which Ethiopia’s HMIS is built, and it complies with a range of industry-recognized standards.

To launch the mHealth initiative, Dimagi—the technology partner of this initiative—drew upon global experience to develop the following mobile applications to the local context:

- HEW app: Supports HEWs in registration, prioritization, referral, and follow-up of RMNCH service delivery and provides automated job aids.
- Health center app: Allows midwives and health workers at the health center level to confirm referrals from HEWs for RMNCH services in the catchment area and share information with HEWs related to referral feedback, information about clients that received services at the health center without notifying the HEWs, and delivery notifications.
- HEW focal person app: Used by supervisors of the HEWs who are health care professionals based at the health center to provide remote technical and programmatic support and follow-up. The HEW focal application is uploaded on a smartphone with a mobile data network. The primary role of the HEW focal person/supervisor is to provide technical support to the HEWs on the HEP packages. Thus, the HEW focal person app was designed based on that person’s roles and responsibilities as described in the HEP implementation manual. The HEP focal person also provides support on HEW app operation, including troubleshooting and maintenance of the devices that need repair.
- Client notification (short message service [SMS]-based): Notifies clients of appointment reminder messages in local languages to their mobile number.

### Application Modules

**FIGURE 2:**
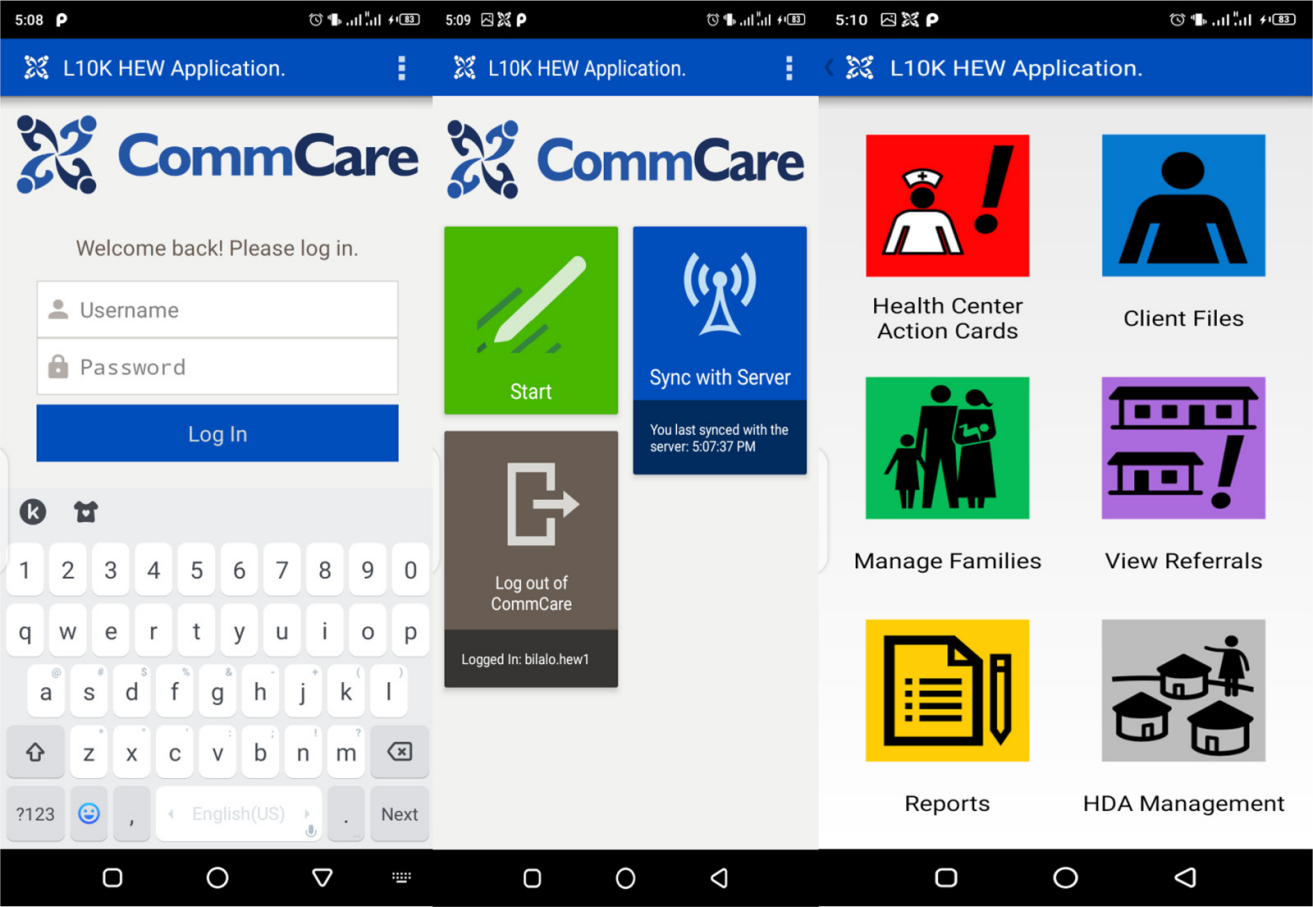
Health Extension Workers CommCare-Based mHealth Application in Ethiopia for Client Registration and Tracking, Screenshots Abbreviations: HDA, Health Development Army; HEW, Health Extension Worker; mHealth, mobile health; L10K 2020, Last Ten Kilometers 2020 Project.

Each HEW was provided a smartphone with the mHealth application to register new clients and track existing clients across the continuum of care (Figure 2). Once logged in, the HEW selects the relevant service or views the list of clients with pending appointments. If a client is not on the list, the HEW initiates a new registration for the client. If a client bypasses the health posts and goes to the health center for services, midwives or health workers in the maternal and child health unit register the client using a tablet-based application, which automatically sends a notification to the HEWs at the health posts with the necessary information, including types of services provided, due date, and place of next appointment.

Since the initiative was implemented in the 4 bigger regions of the country, the interface was developed in different local languages including Amharic, Affan Oromo, and Tigrigna.

#### Reminder, Follow-Up Alert, and Notification

Before the introduction of the mHealth platform, HEWs used a paper folder and tickler box systems to track client appointments for each service type. With the paper-based system, it was cumbersome to trace clients who missed appointments. The mobile applications complement this system by facilitating communication between clients and service providers at different levels because both providers can access updated information on the same client.

The mobile application complements the system by facilitating communication between clients and service providers.

The RMNCH service registration system at the health center triggers mobile notifications for HEWs for the services that require visits at home or the health facility. When a woman or a child receives services at the health facility, their basic information is entered into a database and they are assigned to a specific HEW for follow-up. This information can be entered using a smartphone. These features work online and offline, but whenever a device can connect to the internet, the information is synced to the server. The HEW is then notified of the client who needs a follow-up visit. RMNCH clients (who have mobile phones and registered in the app), HEWs, and midwives receive these notifications via SMS or as an alert through the application on their mobile phones.

If a HEW misses a visit to her RMNCH client at home or health facility level, the system triggers alerts and notifications to be sent to both the HEW and her supervisor about an overdue visit. Notifications and alerts related to the appointment (i.e., due or missed), referral, and births are sent using the mobile app and SMS at both the health center and health post levels.

#### Electronic Job Aids for HEWs

**FIGURE 3:**
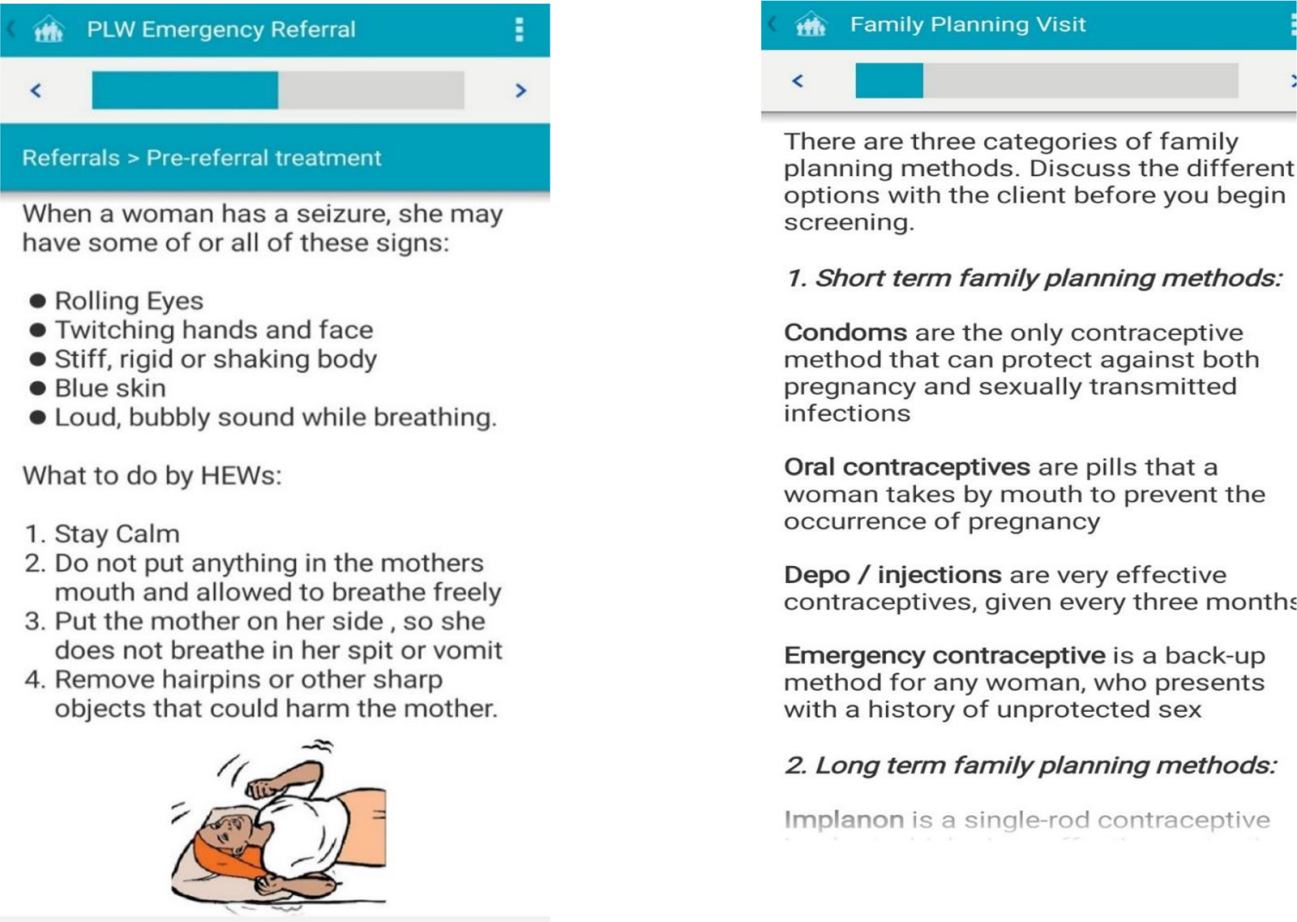
Sample Screenshots of the Electronic Job Aids Included in the CommCare-Based mHealth Application in Ethiopia Abbreviations: HEW, Health Extension Worker; mHealth, mobile health; PLW, pregnant and lactating women.

Job aids and counseling tools were automated for HEWs to ultimately replace the paper-based tools used during household visits and instead use mobile functions such as voice, video or audio clips, and images to enhance the quality and effectiveness of counseling on RMNCH services. Initially, both the paper-based and automated job aids and tools were used simultaneously until the automated system was fully functional. The automated job aids include workflow and protocol support through checklists and decision support; multimedia content played through phones to strengthen counseling and education; and educational messages that could be played through IVR to strengthen counseling and education (Figure 3).

#### Referral and Tracking

Before the introduction of the mHealth platform, HEWs used to provide a paper-based referral slip, which a client would take to the health center. Health center staff would then write a counter referral or feedback slip with relevant information regarding services delivered and any follow-up care needed. This process was automated through the mobile application to strengthen communication and ensure completed 2-way referrals among different providers and facilities.

The referral process was automated through the mobile application to strengthen communication and ensure completed referrals.

To strengthen referral care for mothers and newborns identified with complications during antenatal care (ANC), and postnatal care (PNC) or other visits, a closed user group system was created for HEWs and health centers to be able to call one another within the closed user groups to discuss a case under their care. Contact addresses of caregivers (mobile app users) were registered during the installation of the app with the possibility of updating changes. The providers can give a call and SMS when additional information is needed. Anyone within the closed user group can see ongoing activities and can call others in the group at no cost to themselves. This has enabled more direct communication between health center staff and HEWs.

When a HEW refers a client to the health center designated for health post/HEW using the app, the app automatically triggers a notification to the health center with the necessary referral information, including the planned visit date. If the client is late on the due date, health workers can communicate to the client and/or HEWs with the contact’s address registered in the app. Once the client arrives, the health center application is updated by health workers with the services delivered and whether any follow-up care is required from the HEW. This information is also available on the HEW apps as referral feedbacks and used to track referral status.

## IMPLEMENTATION

**FIGURE 4:**
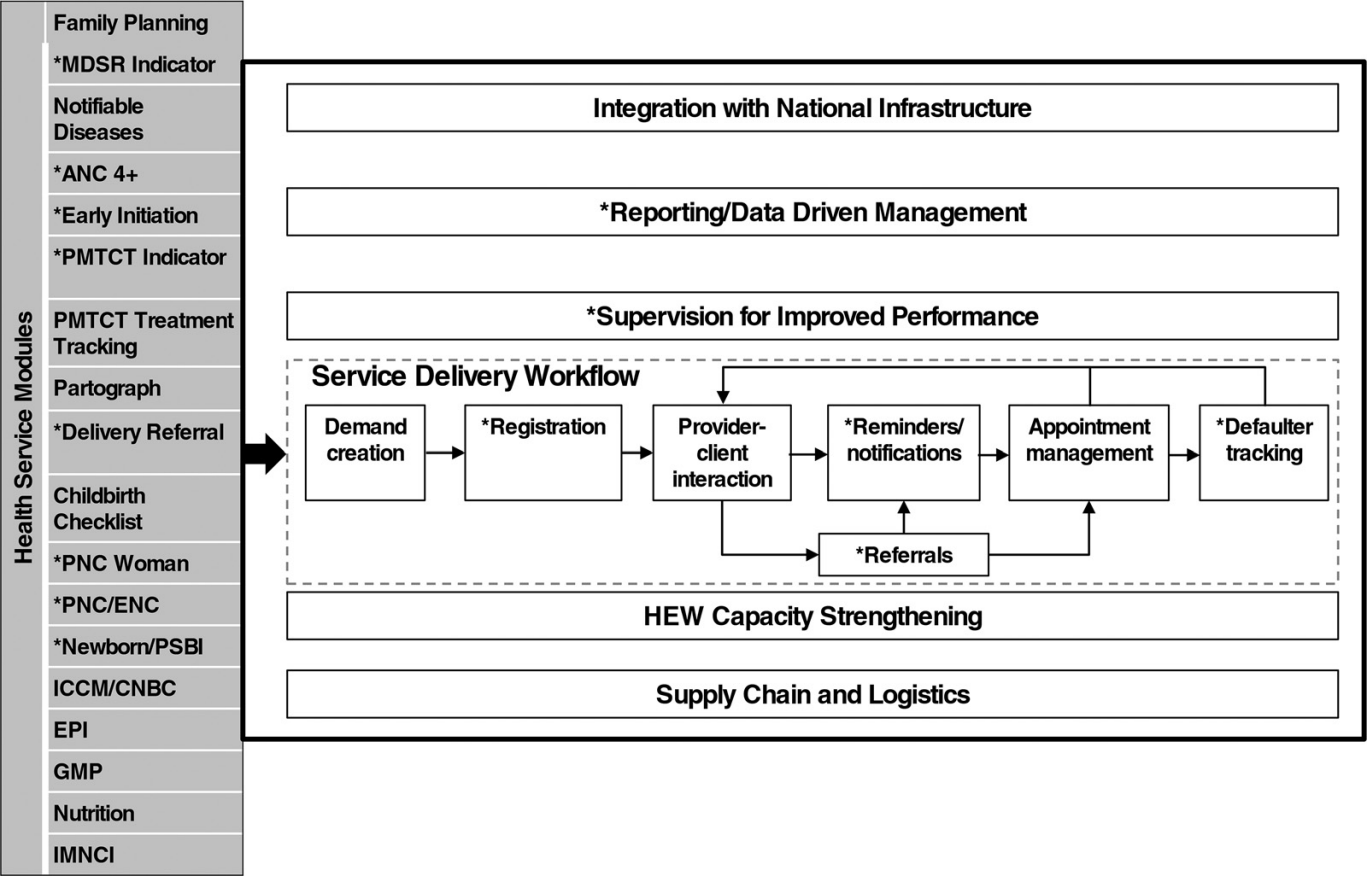
Health Service Modules Prioritized by the Ethiopia Ministry of Health (Left) and the CommCare Platform (Right) Abbreviations: ANC, antenatal care; CBNC, community-based newborn care; ENC, essential newborn care; EPI, Expanded Program for Immunization; GMP, growth monitoring and promotion; HEW, Health Extension Worker; iCCM, integrated community-based case management of common childhood illnesses; IMNCI, integrated management of newborn and childhood illnesses; MDSR, maternal death surveillance and response; PNC, postnatal care; PMTCT, prevention of mother-to-child-transmission (of HIV); PSPI, possible serious bacterial infection.

The mHealth platform was developed over 3 phases to achieve the full scope of L10K 2020 and MOH programmatic objectives (Figure 4). The health services prioritized by MOH are shown on the left, while the activities the platform is capable of supporting are on the right.

The phased release approach intended to test the application with the highest priority health interventions:

**Release 1:** This phase focused on quickly developing and releasing the highest priority modules while building organizational capacity for supporting and scaling the technology. The highest priority modules and activities were ANC, delivery, and PNC service recording, referral, and notification. The mobile tool was designed to meet programmatic needs while responding to the context of users and project stakeholders, the workflows required, and the constraints of the operating environment. Release 1 was developed and pilot-tested in Mirab Azernet Woreda in SNNPR with a population of 59,289 (based on the 2007 population and housing census of Ethiopia); 4 health centers, 19 health posts, and 35 HEWs.

**Release 2:** This phase refined and expanded the L10K 2020 priority modules from Release 1 and included the development of the second set of modules. The subset of modules in the second release included family planning, nutrition, child vaccinations, and maternal tetanus toxoid vaccinations. The pilot was expanded into 3 additional woredas with a total population of 426,159 according to the 2007 national census in 3 different regions, namely, Dembecha, Shabe Sombo, and Weire Leke and Medebay Zana woredas in Amhara, Oromia, and Tigray regions, respectively. These woredas have a total of 18 health centers, 84 health posts, 159 HEWs, and 203 mobile users.

**Release 3:** The module in this phase included integrated community case management and community-based newborn care. In addition to designing these programmatic modules, the team integrated feedback from Releases 1 and 2 to improve the usability of the system.

### Training and Material Distribution to mHealth Users

Midwives, HEWs, and their supervisors based at the health centers providing support to HEWs in the intervention woredas received in-depth mHealth training (Table 2). The training provided orientation on smartphones and tablets, key application features, how to install and uninstall the application, logging in and out, syncing data, the application menu, settings, and navigational functionality. Through demonstration, exercises, and testing, the training focused on using the app to register clients, navigate existing client records, record services, use automated job aids for counseling, create referrals and give feedback, monitor performance, trace defaulters, and track reports. Case scenarios for every application module were designed as practical exercises, with 1 trainer per 5 trainees to provide intensive coaching and support during the practical sessions.

**TABLE 2.** Number of mHealth App Users Trained in the Pilot Sites of L10K 2020, Ethiopia, May to December 2017

**Region**	**HEWs and HWs Trained on mHealth**			
	Training of Trainers	HEWs	Midwives and HEP Supervisors	Total
Amhara	5	58	40	103
Oromia	5	47	31	83
SNNP	6	35	28	69
Tigray	5	88	40	133
Total	21	228	139	388

Abbreviations: HEP, Health Extension Program; HEW, Health Extension Worker; HW, health worker; mHealth, mobile health; L10K 2020, Last Ten Kilometers 2020 Project.

L10K 2020 distributed 308 smartphones to midwives, HEWs, and their supervisors after the training as per the number of HEWs at the health post level but only 1 communal smartphone was distributed to each health center irrespective of the number of midwives. Power banks and solar chargers were also provided for lower-resourced facilities based on findings from the landscape assessment.

### Reporting, Supervision, and Performance Monitoring

**FIGURE 5:**
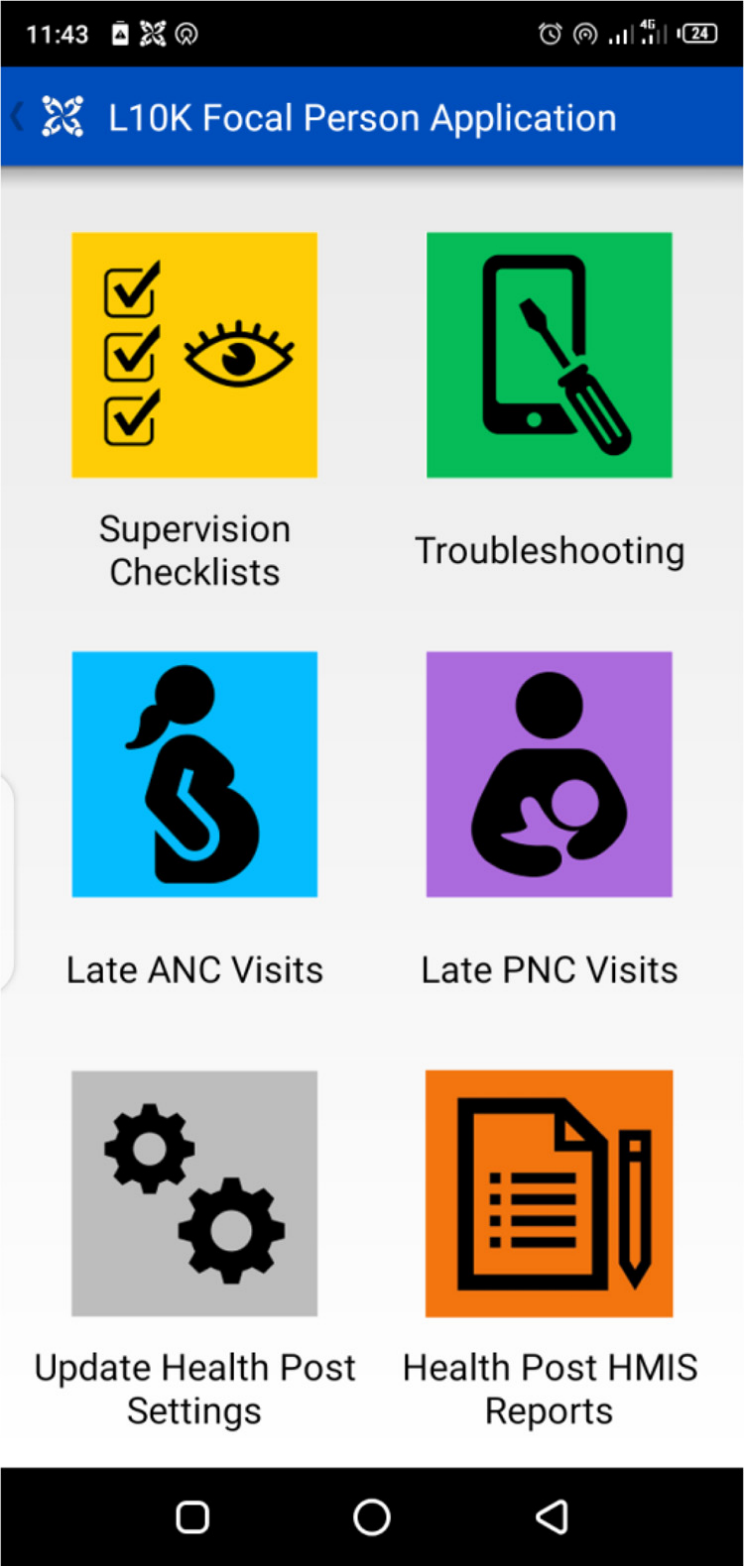
Health Extension Workers (HEWs) Supervisor/Focal Person mHealth Application Screenshots Abbreviations: ANC, antenatal care; HMIS, health management information system; mHealth, mobile health; PNC, postnatal care.

Supervision and performance digital reports were made available to the HEWs and supervisors through the mobile app (Figure 5).

Regular service delivery reports can be generated using the app reporting modules, which were developed to be compatible with HMIS platforms. The app allows HEW supervisors and midwives to see real-time health post performance reports in their catchment through their mobile phones or tablets. These reports were regularly reviewed and discussed with the HEWs to provide immediate feedback and ensure timely action using the mHealth apps.

Weekly and monthly reports (e.g., workers’ activity, daily form submission, completion time), key indicators by facility (e.g., ANC and PNC coverage), and individual-level performance reports for HEWs, focal persons, and midwives were developed and used for routine performance monitoring through the app. App usage was remotely monitored at the national level with a biweekly feedback system for PHCUs and woreda health offices for service provision.

### Troubleshooting

Troubleshooting is a logical and systematic problem-solving approach rather than simply trying things at random. In the mHealth initiative, operational processes for troubleshooting technical issues with the application and with mobile devices were established to manage mobile and app-related problems. Mobile device and application-related bugs were regularly tracked by HEW supervisors. Observations from routine monitoring visits and the usability assessment showed that the most common problems faced by users include malfunctioning charging equipment, phone app crash and lock-out, hardware damage, and mHealth app software uninstalling. A troubleshooting guide was developed and training was provided for HEP supervisors and health sector health information technicians and other program staff at the PHCU and woreda levels. These health workers were able to address most of the common problems using the troubleshooting guide and with remote support from L10K 2020’s regional and central teams. The L10K 2020 teams also provided ad hoc and routine onsite support and supervision to end-users.

## USABILITY AND CONTRIBUTION OF MHEALTH INITIATIVE ON RMNCH SERVICE DELIVERY

L10K 2020 conducted a process evaluation after a year of implementation to identify the implementation strengths and common challenges users faced when adopting the L10K 2020 mobile app system. The study employed purposive sampling techniques to select high- and low-performing app users in the study sites. The study participants include 20 HEWs, 8 midwives, and 6 HEW supervisors.

mHealth app usage and user experiences were evaluated by reviewing system data and key informant interviews of the 34 users. mHealth app usability assessment was also conducted through interviews with the 20 HEWs.

Of 20 HEWs that participated in the usability assessment, 19 preferred the electronic mHealth app over the paper-based client tracking system. Box 1 highlights specific benefits as reported by end-users.

Nearly all the HEWs interviewed on their experience using the mHealth app preferred the app over the paper-based tracking system.

BOX 1 **Benefits Reported in Using the mHealth App in Ethiopia**

- Facilitates remote performance tracking and monitoring
- Enhances quality and timely care for clients
- Provides reliable, quality, and on-time data for action
- Supports access client’s previous and current clinical information
- Strengthens 2-way referral linkages, notifications, and feedback systems with the primary health care unit system
- Provides dynamic job aids to improve clinical skills and client counseling
- Promotes early pregnancy identification and tracking clients in their reproductive, maternal, newborn, and child health care

*Using the paper-based system was difficult to address all eligible clients for services. It was also challenging to access information about pregnant women who received services at the health center and higher health facilities [through self-referral]. Now, we can trace defaulters and follow clients easily, provide timely services, and access information about the clients who bypass our health posts through application notifications and referral linkages. It also saved our time and effort because we are not expected to go to each village for notifying clients about each visit. Instead, we can send a message or call the client or Women’s Development Army member using the mobile phone.* —HEW, 24 years old, Oromia

The major challenges (Box 2 includes details) that were collected from end-users through routine reports, field visits, and evaluation were reported to the project management as part of the project’s monitoring and follow-up and for timely decision making to correct problems. One of the challenges was the high turnover of trained HEWs and health workers. About half of the HEWs trained on the mHealth app left their duty station for different reasons including resignation, education opportunities, or transfer within a year of the implementation period. There were also very few health information technicians to handle the fluctuating demand for ICT support to fix mobile devices and mHealth application bugs. Users worried about losing their mobile devices, and fear of damage or loss meant they sometimes left the devices at home during a community visit. From a total of 308 smartphones distributed for mHealth users, about 7 (3%) tablets were reportedly lost, and 86 (28%) were damaged within the first year of pilot implementation.

BOX 2 **Challenges Reported in Using the mHealth App in Ethiopia**

- Work overload when using the app and the existing paper-based recording system
- Fear of theft and loss of phones and tablets
- Burden of carrying multiple high-value devices
- Shortages of phone and tablets require sharing devices across service delivery points
- Delays in repairing or replacing equipment
- Use of mobile devices for other purposes (e.g., video recording, installing other mobile apps), which can affect the app’s functionality
- Delays in reporting nonfunctional device/app and untimely maintenance
- Interruptions of mobile data connection and unavailability of consistent mobile airtime

Each health center received a single mobile device that needed to be used across multiple service delivery points (i.e., ANC, delivery, PNC, child health), which limited usage by health facility staff. As a result, health center app users—especially midwives who used communal devices–used different strategies to reduce clients’ waiting time during service provision such as through exchanging the device based on caseload across the different service delivery points, assigning a focal person to register cases using the app, transferring cases from registration book to the app, doing a daily audit of clients that received services, and recording missed data in the app.

HEWs reported that although solar chargers and power banks were provided for low-resource health posts, they did not always work.

*It is difficult to have the risk of mobile device or tablet loss. We [also] have no power source at the health post and usually, we send the mobile devices through another person for charging to the nearby town. We also travel a long distance alone for outreach services or home visits with two mobiles, including our phones. In such a situation, we worried a lot not to lose them … and we usually left the mobile device at home though it is very important for our work.* —HEW, 30 years old, Tigray

### Usability of the mHealth Solution

Sixteen of the 20 HEWs who used the mHealth app reported that it was easy and enjoyable to use and they understood its intent, purpose, and potential for impact in their job. HEWs who were engaged in the usability study rated core features of the application as “easy” to “very easy” after being asked to simulate application use in real-life situations. Despite the majority of users (80%) having no prior experience with smartphones or apps in their personal lives, users were generally confident in finding the app on the phone and completing basic form navigations. This suggests that a user’s lack of familiarity with smartphone technology is not necessarily a key barrier to increasing app usage.

Despite 80% of users having no prior experience with smartphones or apps, users were generally confident in finding the app on the phone and completing basic form navigations.

Many mHealth users also demonstrated a general understanding of how the app could improve the efforts of HEWs and health centers in case management. HEWs demonstrated high literacy levels and general comfort in reading text questions and counseling guides on the small phone screen. Many users also demonstrated intuitive ease in playing the audio recordings associated with counseling messages.

### Standardized and Quality Service Delivery

mHealth users reported that they used the app as a job aid and a comprehensive tool to record and send data, monitor services, and exchange referral and reminder messages and notifications. HEWs explained that they used the mHealth application as a job aid and counseling guide during service provision. They reported that they believed clients’ adherence to the pregnancy continuum of care improved as a result of standardized care and counseling services. mHealth application users also described the app as a useful tool that helped them improve interactions, linkages between facilities, and timely exchange of real-time information and achieve higher quality provision of standardized services.

*mHealth enables us to have real-time information and deliver quality and timely services for pregnant and post-natal women. The paper-based system takes time and extra effort to get the previous history of the clients about specific services, but this mHealth app enabled us to access all types of information about the clients. We used it as a checklist for our services. This simplified our routine job more than ever on the provision of standardized care for clients.* —HEW, 26 years old, Oromia

### Improved Linkage and Real-Time Information Exchange at the PHCUs

The mHealth strategy bridged communication gaps between health care workers and HEWs. It assisted HEWs to more easily identify pregnancy danger signs and complications, leading to more timely referrals through the app’s electronic forms.

The mHealth strategy bridged communication gaps between health care workers and HEWs and helped HEWs to more easily identify pregnancy danger signs and complications.

Users reported that the mHealth app helped HEWs and health workers to exchange information and to monitor each client’s appointment dates, location, and types of RMNCH services. The app also helped them to easily identify and trace defaulters and bring them back into care.

*With a paper-based system, it was difficult to know and retrieve late users for RMNCH services, especially the areas with scattered populations and challenging topography, but now we can trace them and can provide services per the schedule. It also supports us to pay attention to when services need to be scheduled services and improved our interaction with clients to improve the coverage of MNCH.* —HEW, 28 years old, Amhara

### Pregnancy Registration and Adherence to Treatment Protocols and Continuity of Care

**FIGURE 6:**
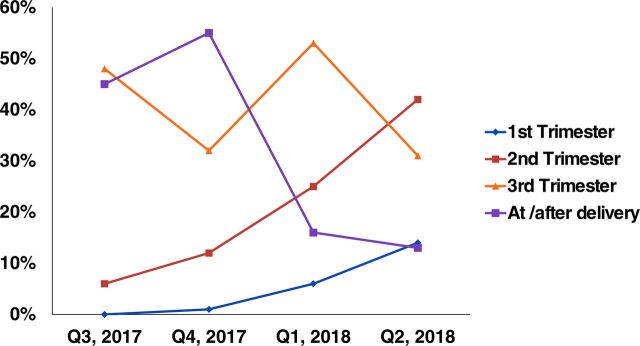
Increased Early Pregnancy Registration as a Result of the App at L10K 2020 mHealth Pilot Project Sites in Ethiopia, July 2017–June 2018 Abbreviations: mHealth, mobile health; L10K 2020, Last Ten Kilometers 2020 Project.

Early identification and registration of pregnant women is a critical entry point to providing focused ANC, delivery, and PNC services. At the beginning of the mHealth initiative, the majority of the pregnant women were registered by HEWs in the third trimester and or later. Data from the mHealth app showed an increase in pregnant mother registration by the HEWs in the first and second trimesters at mHealth PHCU pilot sites as a result of case referrals and notification through the app (Figure 6).

Key indicators of the continuum of maternal and child care services such as ANC, delivery assisted by a skilled birth attendant, and PNC services showed a good level of adherence to RMNCH care-seeking in the implementation period. Among births registered in mHealth apps, 83% of clients were able to receive PNC, with 28% of these clients receiving it within 2 days of giving birth. This better coverage in the implementation period as compared with the previous trend in the woreda might be due to the use of the mHealth apps for birth notification and a reminder that improves the tracking system for services in the pregnancy continuum of care. The below quote corroborates this finding.

*The app helped us to remotely follow HEW performance. We have access to see all client information in our catchment area who registered in the app, such as clients' due dates for ANC, delivery, and PNC. We can identify clients who are late in receiving those services, the reason for the delay, and take timely corrective measures by discussing with the HEW. Our coverage has improved as a result of our close follow-up through the app and gives us room to quickly fix problems.* —HEW supervisor, 24 years old, Amhara

**FIGURE 7:**
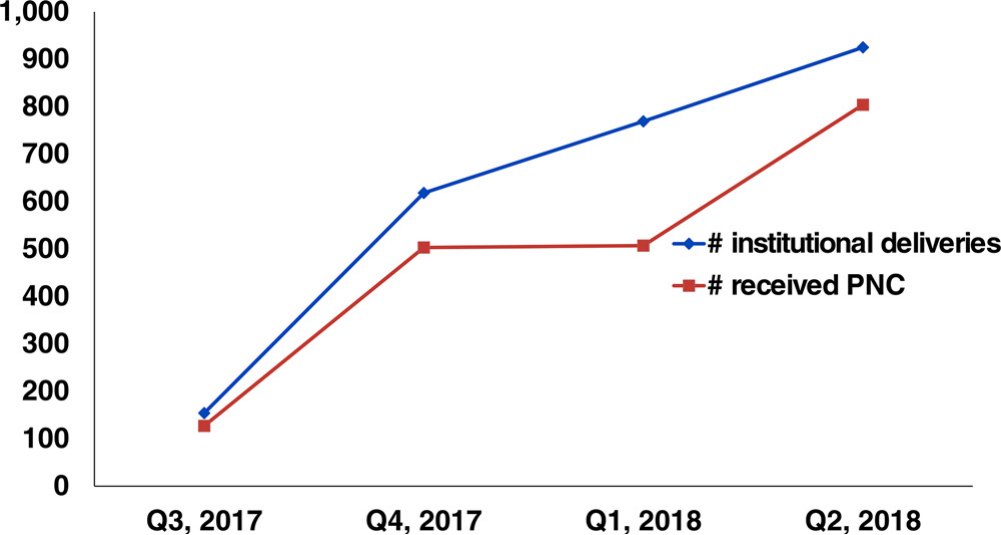
Trends of Delivery and Postnatal Care in mHealth Pilot Sites in Ethiopia, 2017–2018 Abbreviations: mHealth, mobile health; PNC, postnatal care.

In Ethiopia, most women discontinued receiving care at the postpartum stage.^6^^,^^13^ Most women were receiving PNC services after receiving delivery care, and this could be due to the program effect (Figure 7).

## mHealth App used at scale for eCHIS program

The mHealth app was designed to be incorporated into the envisioned eCHIS plan of the country’s health system. Realizing the learning from the implementation of the mHealth initiative and its potential and wide applicability, the MOH took over the mHealth platform including the development of further programmatic modules and ongoing maintenance for large-scale implementation of the national eCHIS. L10K 2020 worked with MOH to adapt the existing mHealth tools to meet the requirements of the eCHIS and is supporting MOH to scale up the eCHIS to 8,000 more HEWs by 2025 to improve the coverage and quality of primary care through data-based performance. The eCHIS application digitizes the existing paper-based, manual family folder, and service workflows to record and report household member health and related data. The system captures data on the HEP and other community-level services. Improving HEP performance and community health outcomes was one of the objectives of the system.

Realizing the mHealth initiative’s potential and wide applicability, the MOH took over the mHealth platform, including developing further programmatic modules and maintenance for large-scale implementation of the national eCHIS.

L10K 2020 and Dimagi provided technical support to MOH experts on the deployment and integration process of the mHealth platform to eCHIS. MOH officials and L10K 2020 experts developed a plan to install a locally hosted instance of the mobile platform to run the eCHIS application and build the capacity of local experts to effectively hand over the integration of the eCHIS system. Major activities accomplished by L10K 2020 during the transition to eCHIS included: (1) local installation of the mobile platform, (2) capacity building of local experts on server set-up and use of the front-end features, and use and customization of the mobile android application; and (3) content review of the eCHIS family folder module. L10K 2020 facilitated national and regional master technical and management training of trainers, supported cascading of the training to end-users, and procured and distributed approximately 1,150 tablets for eCHIS end-users at health posts. As of March 2020, the eCHIS app had been implemented at 1,386 health posts in 134 agrarian woredas, with the MOH planning to scale it nationwide.

## LESSONS LEARNED

L10K 2020’s health sector stakeholder engagement across levels when developing, testing, and deploying the mHealth applications was critical to effectively cultivating ownership as well as ensuring skills and knowledge transfer at all levels. Smooth handover and fully-scaled government use depended on a sound, cocreated strategy to sustainably build local capacity, develop clear operational troubleshooting and ICT-supported guidance tools, and iterating implementation based on experience. The landscape assessment was the most important factor in understanding opportunities, resources, and barriers affecting planning and benchmarking toward maximizing the use of the app and integrating it with the nascent eCHIS vision.

The landscape assessment was the most important factor in understanding opportunities, resources, and barriers affecting planning and benchmarking toward maximizing the use of the app and integrating it with the eCHIS vision.

Despite the numerous benefits of the mHealth initiative, continued success will require vigilance in consistently mitigating the following challenges:

- There was a critical shortage of infrastructure such as mobile data capacity, electricity, smartphones and tablets, and solar chargers to effectively leverage the benefits of mobile technology. Gaps identified through routine monitoring include delayed distribution of mobile cards, delayed replacement of damaged or lost mobile devices, and failure in timely reporting of nonfunctional devices or apps.
- mHealth app implementation at the intervention sites was strained by technology infrastructural challenges such as limited data transfer capacity due to weak internet signal, particularly for the variety of mobile devices used among end-users.
- Protracted and widespread internet connectivity interruption due to a national mobile data blackout during several months of piloting the mHealth initiative affected the quality and timely information exchanges within the PHCUs.
- HEW supervisors and program managers were not closely monitoring the service delivery data entered into the application or using these data to make decisions.

Hence, addressing these issues would reduce individual usage disruptions and engender users’ confidence in, reliance upon, and consistent use of the app’s sound capabilities. Also, creating ownership and close monitoring while integrating with the existing system is a much-needed effort because the use of mHealth tools by itself could not incentivize the health system.

## Footnotes

* CommCare (www.commcarehq.org) is an open source, easily customizable, and widely adopted mobile platform that supports frontline workers (FLWs) to track and support their clients with registration forms, checklists, SMS reminders, and multimedia in low-resource settings. CommCare was used as solution based on prior use in the country and opinion of experts that it can be easily customized to fit in to MOHs future plan. As such we would like to acknowledge that it is the product of Dimagi.

## Notes

**Peer Reviewed**

**First published online:** July 29, 2021.
